# Analysis of a Large Standardized Food Challenge Data Set to Determine Predictors of Positive Outcome Across Multiple Allergens

**DOI:** 10.3389/fimmu.2018.02689

**Published:** 2018-11-27

**Authors:** Sayantani Sindher, Andrew J. Long, Natasha Purington, Madeleine Chollet, Sara Slatkin, Sandra Andorf, Dana Tupa, Divya Kumar, Margaret A. Woch, Katherine L. O'Laughlin, Amal Assaad, Jacqueline Pongracic, Jonathan M. Spergel, Jonathan Tam, Stephen Tilles, Julie Wang, Stephen J. Galli, Kari C. Nadeau, R. Sharon Chinthrajah

**Affiliations:** ^1^Sean N. Parker Center for Allergy and Asthma Research, Stanford University School of Medicine, Stanford, CA, United States; ^2^Department of Pharmacy, Lucile Packard Children's Hospital Stanford, Stanford, CA, United States; ^3^Department of Medicine, School of Medicine, Stanford, CA, United States; ^4^Division of Allergy and Immunology, Cincinnati Children's Medical Center, Cincinnati, OH, United States; ^5^Division of Allergy and Immunology, The Ann and Robert H. Lurie Children's Hospital of Chicago, Chicago, IL, United States; ^6^Division of Allergy and Immunology, The Children's Hospital of Philadelphia Department of Pediatrics, Perelman School of Medicine at University of Pennsylvania, Philadelphia, PA, United States; ^7^Division of Clinical Immunology and Allergy, Children's Hospital Los Angeles, Los Angeles, CA, United States; ^8^ASTHMA Inc. Clinical Research Center, Northwest Asthma and Allergy Center, University of Washington, Seattle, WA, United States; ^9^Division of Allergy and Immunology, Department of Pediatrics, Icahn School of Medicine at Mount Sinai, New York, NY, United States; ^10^Department of Pathology, Stanford University School of Medicine, Stanford, CA, United States; ^11^Department of Microbiology and Immunology, Stanford University School of Medicine, Stanford, CA, United States

**Keywords:** food challenge, cumulative tolerated dose, AUC, biomarker evaluation, time-dependent ROC

## Abstract

**Background:** Double-blind placebo-controlled food challenges (DBPCFCs) remain the gold standard for the diagnosis of food allergy; however, challenges require significant time and resources and place the patient at an increased risk for severe allergic adverse events. There have been continued efforts to identify alternative diagnostic methods to replace or minimize the need for oral food challenges (OFCs) in the diagnosis of food allergy.

**Methods:** Data was extracted for all IRB-approved, Stanford-initiated clinical protocols involving standardized screening OFCs to a cumulative dose of 500 mg protein to any of 11 food allergens in participants with elevated skin prick test (SPT) and/or specific IgE (sIgE) values to the challenged food across 7 sites. Baseline population characteristics, biomarkers, and challenge outcomes were analyzed to develop diagnostic criteria predictive of positive OFCs across multiple allergens in our multi-allergic cohorts.

**Results:** A total of 1247 OFCs completed by 427 participants were analyzed in this cohort. Eighty-five percent of all OFCs had positive challenges. A history of atopic dermatitis and multiple food allergies were significantly associated with a higher risk of positive OFCs. The majority of food-specific SPT, sIgE, and sIgE/total IgE (tIgE) thresholds calculated from cumulative tolerated dose (CTD)-dependent receiver operator curves (ROC) had high discrimination of OFC outcome (area under the curves > 0.75). Participants with values above the thresholds were more likely to have positive challenges.

**Conclusions:** This is the first study, to our knowledge, to not only adjust for tolerated allergen dose in predicting OFC outcome, but to also use this method to establish biomarker thresholds. The presented findings suggest that readily obtainable biomarker values and patient demographics may be of use in the prediction of OFC outcome and food allergy. In the subset of patients with SPT or sIgE values above the thresholds, values appear highly predictive of a positive OFC and true food allergy. While these values are relatively high, they may serve as an appropriate substitute for food challenges in clinical and research settings.

## Background

During recent years, the prevalence of IgE-mediated food allergies has steadily increased and has emerged as a significant health crisis (1) affecting 8% of the pediatric population with more than 30% of these children with multiple food (multifood) allergies (2). Not only are childhood food allergies associated with comorbid atopic conditions such as atopic dermatitis, asthma, and allergic rhinitis, but are also associated with impaired quality of life (3–8).

The diagnosis of food allergy is highly complex (9, 10). Skin prick testing (SPT) and food allergen-specific immunoglobulin E (sIgE) are commonly used to determine allergenicity, however outcomes are often variable. High thresholds of both SPT and sIgE have been established for specific foods and tend to correlate with reactivity, such as sIgE > 15 KU/L and SPT > 8 mm associated with 95% positive predictive value (PPV) for tree nuts (11). However, thresholds are less useful for intermediate values where there is already a doubt whether the patient is truly allergic (12–21), and may be associated with false positives (10, 22). Children in particular have a higher rate of sensitization without true allergy (23). Other biomarkers that have been explored include basophil activation tests (BATs) as well as the measurements of allergen-specific IgG, total IgE (tIgE), and component resolved diagnostics, but definitive thresholds remain to be established (24). Due to these limitations, the current gold standard for confirming food allergy is the double-blind, placebo-controlled food challenge (DBPCFC) (9, 10), which is typically performed in the research setting as part of inclusion into clinical trials; however, DBPCFCs are not without a number of limitations. While food challenge guidelines have been recommended in the literature, dosing strategies are not allergen-specific (25). DBPCFCs require multiple days of challenges for multi-food allergic individuals, which can significantly increase the cost. The most significant limitation is that food challenges carry the risk of potentially inducing severe anaphylaxis which may require hospitalization or care in the intensive care unit (26).

In this paper, we attempt to identify potential prognostic indicators for multi-food allergic individuals associated with outcomes during oral food challenges (OFCs) which could aid in risk stratification for designing challenge protocols for clinical trials. We tested data obtained from eligible participants from several food allergy trials that required either baseline DBPCFCs or unblinded food challenges as an inclusion criteria. In our analysis, we attempt to identify factors that may better predict food allergy outcomes in the research and clinical setting and provide guidance toward dosing strategies.

## Methods

### Data source

All clinical trial participant data from food allergy studies conducted under IRB approved protocols were entered into a standardized database. The database was created by a board certified Allergy/Immunology physician and all food challenges were conducted, evaluated, and documented by trained research clinicians. Data entry was performed by trained research staff. Quality checks of data were performed by our data entry and statistics team.

### Skin prick tests, IgE blood tests, and oral food challenges

Between September 2010 to March 2016, participants were recruited to undergo OFCs as part of screening for clinical trial enrollment at 7 sites under an Investigational New Drug (IND) at Stanford University. During the initial screening visit, SPT and IgE values were obtained for each participant in the clinic at the time of the visit or from previous testing at a physician's office, depending on clinical trial inclusion criteria. SPT consisted of a positive histamine control, a negative saline control (both from Hollister-Stier) and allergen extracts from Greer. SPTs were performed on the volar surface of the forearm or back after application of the respective allergen solution. Mean wheal diameter was measured after 20 min. Allergen-specific IgE levels were measured by ImmunoCAP fluorescence enzyme immunoassay. Challenges to each food allergen were performed only in participants with suspected food allergy, defined broadly as an sIgE > 0.35 kU/L and/or a positive SPT (>3 mm above the negative control) to the challenged allergen. OFCs were standardized in methodology and escalated to at least 500 mg cumulative food protein to each of the participants' suspected allergens. Participants with previous reactions to food requiring the use of epinephrine for adverse reactions were eligible for screening and challenges under each study; however, those with a past history of intubation or hypotension related to a food allergy were excluded.

While most of the included challenges were conducted as DBPCFCs, some challenges were unblinded OFCs. All food challenges included for the purpose of analyses will be referred to as OFCs, herein, regardless of blinded status. Excluding such differences in blinding, all OFCs were performed using standardized methodology with respect to monitoring, according to validated guidelines (10, 27, 28). Challenges to eleven different food allergens were included in the analyses, consisting of almond, cashew, egg, hazelnut, milk, peanut, pecan, pistachio, sesame, walnut, and wheat. Typically challenges started with as small as 1 mg (for pistachio), then 2, 5, 20, 50, 100, 100, 100, 123 mg (for pistachio) or 124 mg. Pistachio started at 1 mg due to safety concerns since only those positive to a cashew challenge, were also challenged to pistachio. Challenges to allergens other than those mentioned above were defined as “other” and excluded from further analyses given the limited number of challenges performed to such allergens. Each OFC consisted of sequentially escalating doses of food protein ingested by the participant every 15 min as tolerated. Food protein was administered in flour form mixed in an appropriate vehicle, such as applesauce or pudding. During the course of the challenge, vital signs and pertinent physical examinations were repeated at least every 15 min at the discretion of the clinician. Type and severity of each dose-related allergic adverse event were determined and classified according to Bock criteria (27), and participants tolerating 500 mg cumulative protein dose during the challenge were considered to have a negative challenge, for the purpose of analysis. Cumulative tolerated dose (CTD) was defined as the last ingested cumulative protein dose at which no dose-related allergic adverse event occurred. All aspects of the studies from which data was obtained were authorized by the IRBs at each site.

### Statistical analysis

Challenges were censored at 500 mg CTD if the challenge was negative. A cumulative incidence plot and median survival were reported by food, and the equality of the incidence curves was tested using the log-rank test. The survfit function of R's survminer package was used to fit the model (29).

To determine possible predictors of a positive challenge, Cox proportional hazards models containing Gaussian random effects (i.e., frailty models) were fit to the primary outcome as a function of each clinical and demographic feature, adjusting for challenge food with a random effect for participant. The coxme function was used to fit each model (30). Hazard ratios and 95% CIs were reported.

To determine thresholds of SPT, sIgE, and the sIgE to tIgE ratio (IgEr) that best discriminated challenge outcome, the OptimalCutpoints package was implemented using receiver operator characteristic (ROC) curves based on Youden's index (31, 32). Next, a logistic regression model was fit to both SPT and sIgE then to SPT and sIgEr for each food. The ModelGood package was used to calculate the AUC from each multivariable model (33). The set of 5 ROC analyses were compared for each food graphically and by AUC.

To incorporate the dose-varying nature of the food challenge outcome, a dose-dependent ROC was used, predicting the probability of a positive challenge to a maximum cumulative dose of 500 mg. The survivalROC package was used to determine the optimal threshold, while time ROC was used to calculate the AUC, PPV, and negative predictive value (NPV) at the determined threshold by dose (34, 35). Kaplan-Meier curves were plotted based on the determined threshold, and *P*-values from the log-rank test were reported. Within positive OFCs, concordance of SPT and sIgE thresholds and SPT and sIgEr thresholds for each food was assessed and accuracy was reported.

In order to compare the two ROC methods, AUCs were derived from 1,000 bootstrap samples per ROC method, allergen, and marker. We then took the difference in the two AUCs and calculated a 95% confidence interval around the difference.

All analyses were conducted at the 0.05 alpha level. *P*-values were not adjusted where multiple comparisons were made. Analyses were conducted using R v.3.4.3 (36).

### Data management

Any value of sIgE > 100 kU/L was truncated to 101 due to clinical lab processing. If SPT or sIgE were not performed during screening then previously collected SPT and/or sIgE available within 12 months of the OFC were included in the analysis (14). Negative control (saline) SPT was subtracted from the raw food SPTs prior to analysis. Any SPT that was collected after the food challenge or collected more than 12 months before the challenge was excluded. If a subject had more than one value for SPT or sIgE, then the value obtained most recently was used.

**Figure 1:**
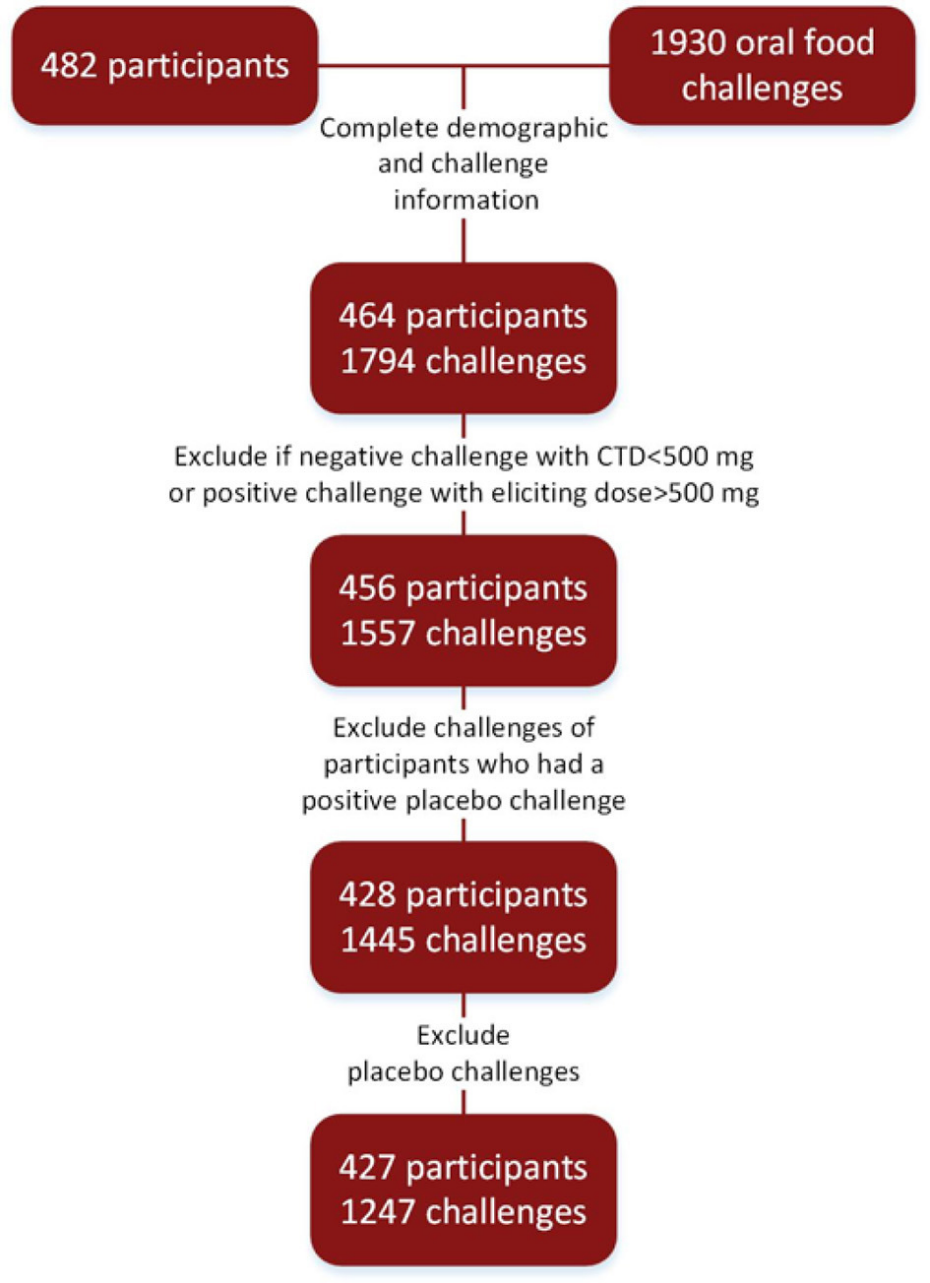
Consort diagram: food challenges conducted in research settings to arrive at final cohort.

To account for differences in maximum challenge doses, positive challenges with CTDs of 500 mg protein or higher were re-coded as having negative challenges. Subjects who had unknown or non-reported ethnicity were coded as missing ethnicity. Subjects with race of Native Hawaiian, other, or not reported were coded as other. Challenges to oat (placebo) were excluded from analyses. Further, challenges reported as negative with CTDs of < 500 mg cumulative protein were also excluded. Placebo challenges were not included in the analysis. A consort of these steps is illustrated in Figure 1.

## Results

### Baseline demographics

Four hundred and twenty-seven participants were challenged to at least one food (Figure 1). Ages ranged from 1 to 54, with a median age of 9 years. The cohort was comprised of mostly non-Hispanic (97%), Caucasian (61%), and males (61%). The majority of participants also had atopic history, including asthma (62%), allergic rhinitis (77%), and atopic dermatitis (AD) (73%). The median number of doctor diagnosed food allergens was 5, with only 2% of the cohort being mono-food allergic. The median tIgE was 491 kU/L (Table 1).

**Table 1:** Baseline demographics.

**Characteristic**	Total* (*n* = 427)
Age in years, median (range)	9 (1–54)
Male	259 (61%)
Non-Hispanic	406 (97%)
**RACE**	
Caucasian	259 (61%)
Black	6 (1%)
Asian	109 (26%)
Multiracial	42 (10%)
Other	5 (1%)
**ATOPIC HISTORY**	
Asthma	240 (62%)
Allergic rhinitis	294 (77%)
Atopic Dermatitis	279 (73%)
Number of food allergens, median (range)	5 (1–16)
Mono-food allergic	10 (2%)
Total IgE (IU/L), median (range)	491 (17.8–3,366.0)

* *Count and percent of total subjects unless otherwise noted*.

### Challenge overview

Eighty-five percent of OFCs resulted in a positive outcome. Between 41 and 100% of all OFCs conducted across foods were positive (Table 2). For instance, all pistachio challenges had positive outcomes, however only cashew allergic participants were challenged to pistachio. Cashew and pecan challenges had the next highest percent of positive challenges (93%), followed by peanut (92%). Some participants repeated food challenges to the same food allergen over time, therefore the number of positive OFCs may be higher than the number of unique allergic participants. The largest number of food challenges were conducted for peanut (*n* = 377) with 77% of participants having positive challenges. Only 41% of almond challenges resulted in a positive challenge outcome.

**Table 2:** Challenge summary by allergen.

**Allergen**	**Positive OFCs/total OFCs (%)**	**Allergic participants (%)**
Almond	30/73 (41)	29 (7)
Cashew	151/163 (93)	150 (35)
Egg	63/71 (89)	60 (14)
Hazelnut	68/102 (67)	65 (15)
Milk	67/77 (87)	66 (15)
Peanut	347/377 (92)	330 (77)
Pecan	88/95 (93)	88 (21)
Pistachio	60/60 (100)	59 (14)
Sesame	30/42 (71)	30 (7)
Walnut	121/138 (88)	120 (28)
Wheat	13/16 (81)	13 (3)
Other	16/33 (48)	13 (3)
Total	1054/1247 (85)	410/427 (96)

*Oral food challenge (OFC); Other foods consisted of barley, brazil nut, chickpea, crab, garbanzo bean, macadamia, mustard, rye, shellfish, and soy. Seventeen subjects did have a positive challenge to any food tested. Allergic is defined as the number of participants who had a positive challenge to that food. Participant may have had repeat challenges to the same food*.

**Figure 2:**
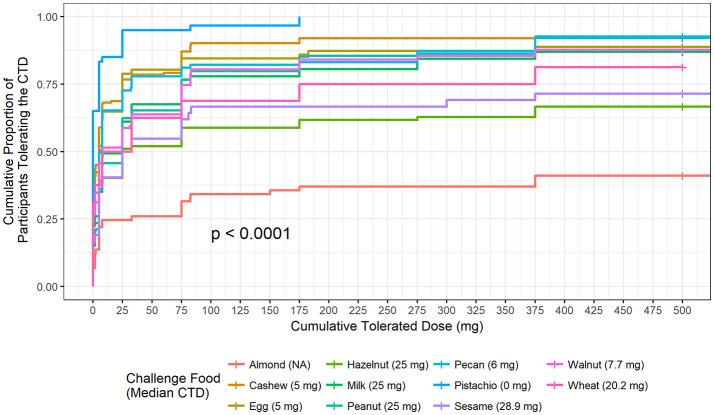
Cumulative tolerated dose by food. Each food is plotted to understand the proportion of participants who tolerated specified cumulative tolerated doses (CTD). Median CTD is the highest cumulative dose at which 50% of participants had no dose-related allergic reaction and is listed in the figure legend in parenthesis.

The highest median CTD at which 50% of participants had no allergic reaction was 28.9 mg (for sesame), while the other challenged foods had lower median CTDs; except for challenges to almond where < 50% of participants had a positive outcome (Figure 2). No participant challenged to pistachio in our Center tolerated a cumulative protein dose >175 mg and 50% reacted at the first dose (CTD median = 0).

Average SPT values in the cohort ranged from 6.2 mm for almond to 13.6 mm for cashew and peanut (Table 3). Peanut had the highest median sIgE (67.55 kU/L) followed by wheat (61.5 kU/L) and almond had the lowest (4.39 kU/L).

**Table 3:** Biomarker summaries per specific food.

**Food**	***n***	SPT*, mm	***n***	**sIgE, kU/L**	***n***	**sIgEr**
		**Mean (range)**		**Median (range)**		**Median (range)**
Almond	42	6.2 (0–21.5)	52	4.39 (0–101)	36	0.008 (0–0.085)
Cashew	106	13.6 (0–30.5)	114	10.85 (0–101)	79	0.028 (0.001–0.249)
Egg	43	10.8 (2.5–22.5)	36	10.14 (0.75–100)	28	0.024 (0.002–0.430)
Hazelnut	70	8.9 (0–35.5)	77	11.30 (0–100)	61	0.026 (0.001–0.422)
Milk	53	12.2 (0–26.0)	58	14.55 (0–101)	46	0.029 (0–0.437)
Peanut	302	13.6 (0–33.0)	268	67.55 (0–101)	168	0.080 (0–0.452)
Pecan	64	9.7 (0–21.0)	63	8.30 (0–101)	53	0.018 (0.002–0.200)
Sesame	23	11.2 (0–27.5)	29	9.98 (0–100)	24	0.027 (0–0.209)
Walnut	92	10.0 (0–26.0)	101	13.30 (0–101)	80	0.035 (0–0.347)
Wheat	12	8.3 (0–13.5)	13	61.50 (3.30–101)	12	0.068 (0.012–0.410)

* *Subtracting out the negative control*.

Participants with a lifetime history of AD had 1.23-fold higher risk of a positive challenge outcome compared to those without a history of AD (hazard ratio [HR]: 1.23, 95% confidence interval [CI]: 1.00, 1.52) (Table 4). The risk of a positive challenge outcome increased by 4% for every additional doctor diagnosed food allergy (HR: 1.04, CI: 1.01, 1.07).

**Table 4:** Univariable associations of positive challenge.

**Characteristic**	**Hazard ratio (95% CI)**	**Challenges included**
Female	1.00 (0.83, 1.20)	1,246
Hispanic	1.44 (0.85, 2.44)	1,228
Race (ref = Caucasian)		1,236
Black	0.82 (0.36, 1.85)
Asian	1.19 (0.97, 1.45)
Multiracial	1.24 (0.92, 1.67)
Other	1.21 (0.58, 2.54)
Atopic History	
Asthma	1.05 (0.87, 1.27)	1,154
Allergic Rhinitis	0.93 (0.75, 1.15)	1,138
Atopic Dermatitis	1.23* (1.00, 1.52)	1,139
Age	0.99 (0.98, 1.01)	1,247
FEV1	1.00 (0.99, 1.01)	744
FEV1 to FVC Ratio	1.71 (0.35, 8.27)	571
Mono-allergic	0.71 (0.37, 1.37)	1,247
Number of diagnosed foods	1.04** (1.01, 1.07)	1,223
IgE Total (log-scale)	0.93 (0.82, 1.05)	621

*Each row corresponds to a single frailty model based on CTD and challenge outcome*.

* *p < 0.05*;

** *p < 0.01*.

*FEVI, The forced expiratory volume in one second; FVC, Forced vital capacity*.

### Logistic ROC for clinical thresholds

The logistic ROC approach resulted in SPT thresholds that ranged from 4.5 mm for wheat to 14.5 mm for egg for predicting a positive OFC, with AUCs ranging from 0.52 to 0.90 (Table 5). The ROC approach using sIgE resulted in thresholds that ranged from 1.2 kU/L for cashew to 52.2 kU/L for wheat, with AUCs ranging from 0.59 to 0.92. AUCs for sIgEr thresholds ranged from 0.65 to 0.89.

**Table 5:** Logistic ROC thresholds for food challenge outcome.

**Food**	**SPT Cutoff, mm**	**sIgE Cutoff, kU/L**	**sIgEr Cutoff**	**SPT + sIgE**	**SPT + sIgEr**
	**(AUC)**	**(AUC)**	**(AUC)**	**AUC**	**AUC**
Almond	12.5 (0.52)	12.4 (**0.68**)	0.002 (0.65)	0.63	0.61
Cashew	5.0 (0.90)	1.2 (0.85)	0.002 (0.85)	**0.93**	0.82
Egg	14.5 (0.65)	10.7 (0.71)	0.018 (0.81)	0.84	**0.88**
Hazelnut	7.5 (**0.74**)	15.4 (0.59)	0.025 (0.68)	0.72	0.58
Milk	8.5 (0.73)	22.8 (0.61)	0.017 (**0.83**)	0.56	0.76^x^
Peanut	9.5 (0.71)	11.4 (0.81)	0.017 (0.81)	0.73	**0.82**
Pecan	7.5 (0.76)	2.1 (**0.92**)	0.011 (0.81)	0.97^x^	0.88^x^
Sesame	11.5 (0.81)	8.8 (0.86)	0.069 (0.76)	**0.88**	0.89^x^
Walnut	7.5 (0.80)	13.9 (0.78)	0.021 (**0.82**)	0.93^x^	0.67
Wheat	4.5 (0.82)	52.2 (0.83)	0.057 (**0.89**)	0.82	1.00^x^

*Based on ROC analysis of challenge outcome. Bolded values indicate the highest AUC across all markers, excluding those with an x. ^x^Denotes quasi-separation/non-convergence of model. AUC from these models may not be informative. Subjects with negative challenge outcomes were not included. SPT, skin prick test; AUC, area under the curve; sIgE, allergen-specific IgE; sIgEr, specific IgE / total IgE ratio*.

**Figure 3:**
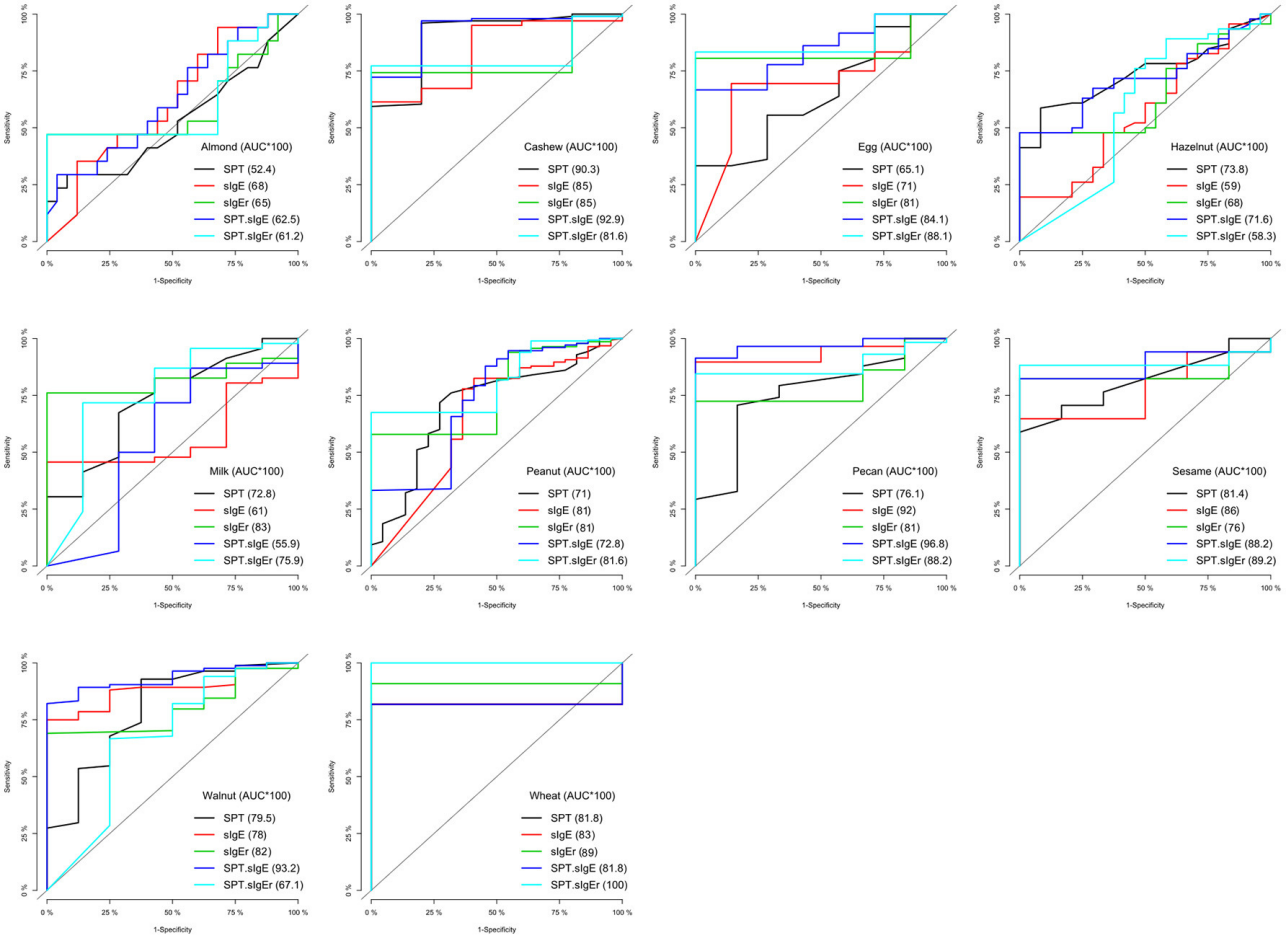
Logistic ROC comparisons of biomarker thresholds by allergen. Comparison of each biomarker to discriminate food challenge outcome. Higher area under the curve (AUC) suggests better discriminative ability. SPT, skin prick test; sIgE, specific Immunoglobin E, sIgEr, ratio of sIgE to total IgE.

In four of the 10 allergens (cashew, egg, peanut, and sesame), the combination of SPT and either sIgE or sIgEr was better at discriminating food challenge outcome than any of the markers individually, and in one instance (for hazelnut), SPT alone was the best (Table 5). For cashew, egg, peanut, and sesame where the joint markers were superior, AUCs were 0.80 and above. A comparison of the joint markers and each individual marker by food are displayed in Figure 3. The best AUC for each food varied between the clinical markers.

### CTD-dependent ROC for clinical thresholds

**Figure 4:**
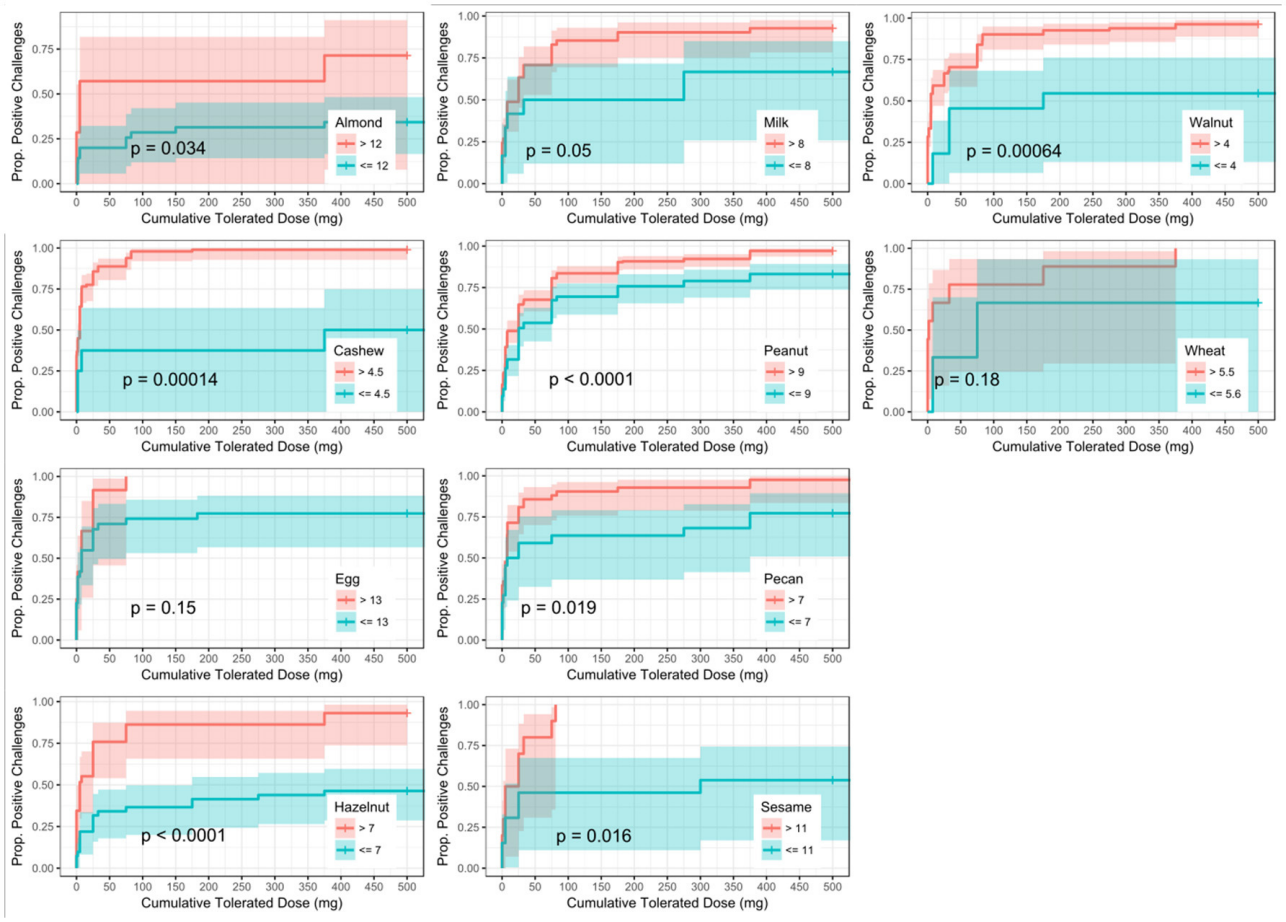
Dose to positive challenge by skin prick test (SPT) threshold and food. Kaplan-Meier curves of dose to positive challenge stratified by the CTD-dependent ROC thresholds for SPT by food. Red lines indicate risk of a positive challenge if SPT is above the threshold, while blue lines indicate risk for participants with SPT at or below the threshold.

ROC analyses were also conducted to assess for CTD and challenge outcome to account for the last tolerated dose in the food challenge outcome. Participants with SPTs above the calculated CTD-dependent thresholds were significantly more likely to not only have a positive challenge, but react at lower doses compared to those with values below the threshold for all foods except milk, egg, and wheat (Figure 4). AUCs for SPT ranged from 0.65 (almond) to 0.98 (cashew) (Table 6). Walnut had the lowest calculated SPT threshold of 4 mm and egg had the highest calculated SPT threshold of 13 mm. While thresholds chosen in the CTD-dependent ROC analysis were similar to those reported for the logistic ROC approach, AUCs were generally higher, though this difference was not significant, in the CTD-dependent ROCs.

**Table 6:** CTD-dependent ROC thresholds at 500 mg CTD.

**Food**	**SPT Cutoff, mm (AUC)**	**PPV**	**NPV**	**sIgE Cutoff, kU/L (AUC)**	**PPV**	**NPV**	**sIgEr Cutoff (AUC)**	**PPV**	**NPV**
Almond	12.0 (0.65^b^)	1	0.68	12.2 (0.71^b^)	1	0.72	0.002 (0.83)	0.68	1
Cashew	4.5 (0.98^b^)	1	0.56	1.2 (0.83^b^)	0.98	0.34	0.002 (0.82^a^)	0.99	0.50
Egg	13.0 (0.67)	1	0.23	9.6 (0.80)	1	0.33	0.012 (0.86^a^)	1	0.30
Hazelnut	7.0 (0.79)	1	0.56	14.6 (0.56)	0.73	0.38	0.022 (0.83)	1	0.59
Milk	8.0 (0.91)	1	0.47	20.1 (0.68^a^)	0.96	0.19	0.016 (0.80^a^)	0.97	0.36
Peanut	9.0 (0.86)	1	0.22	10.7 (0.64^b^)	0.95	0.17	0.017 (0.77^b^)	0.96	0.35
Pecan	7.0 (0.69^b^)	0.95	0.19	1.8 (0.94^b^)	1	0.46	0.011 (0.82)	1	0.14
Sesame	11.0 (0.79^b^)	1	0.46	7.5 (0.40^b^)	0.64	0	0.055 (0.76^b^)	1	0.47
Walnut	4.0 (0.96)	1	0.57	13.5 (0.77)	1	0.24	0.019 (0.87)	1	0.33
Wheat	5.5 (0.90^a^)	1	0.33	43.1 (0.89^a^)	1	0.60	0.027 (0.77^a^, ^b^)	0.88	0.67

*Based on time-dependent ROC analysis for censored survival data*.

a *Could only be estimated at 375 mg*.

b *Had better predictive ability at a lower dose, but AUC at 500 mg (375 mg for wheat) is reported*.

*SPT, skin prick test; AUC, area under the curve; sIgE, allergen-specific IgE; sIgEr, specific IgE / total IgE ratio; PPV, positive predictive value; NPV, negative predictive value*.

**Figure 5:**
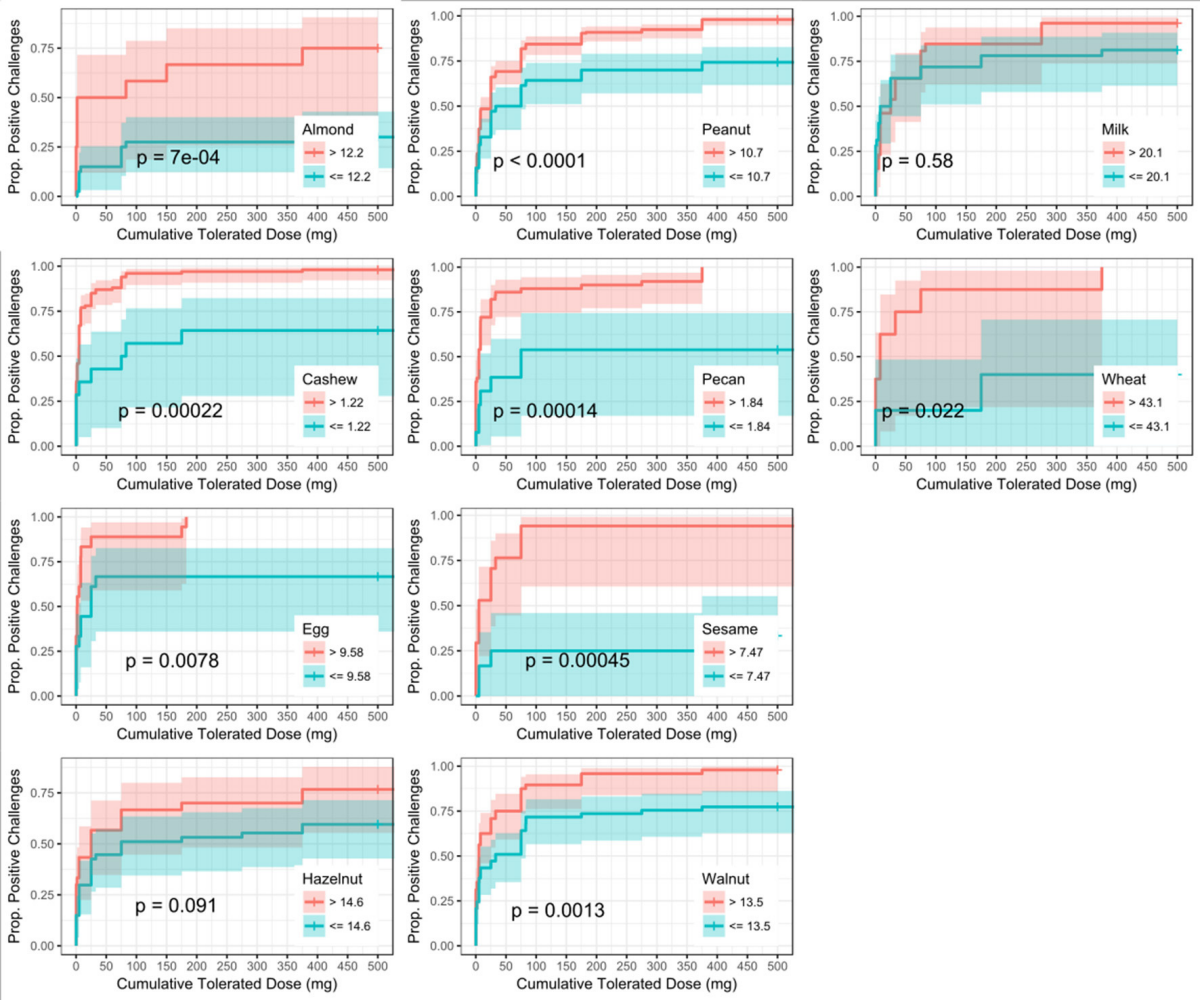
Dose to positive challenge by allergen-specific IgE (sIgE) threshold and food. Kaplan-Meier curves of dose to positive challenge stratified by the CTD-dependent ROC thresholds for sIgE by food. Red lines indicate risk of a positive challenge if sIgE is above the threshold, while blue lines indicate risk for participants with sIgE at or below the threshold.

Similar to SPT, sIgE values above the threshold were associated with a lower dose to a positive outcome compared to those with values at or below the threshold (Figure 5). Cashew had the lowest calculated sIgE threshold of 1.2 kU/L, and wheat was the highest at 43.1 kU/L (Table 6). Cashew, pecan, and wheat thresholds had AUCs above 0.80. Hazelnut and sesame had the lowest AUCs. Threshold values were similar to those chosen through the logistic ROC analysis.

**Figure 6:**
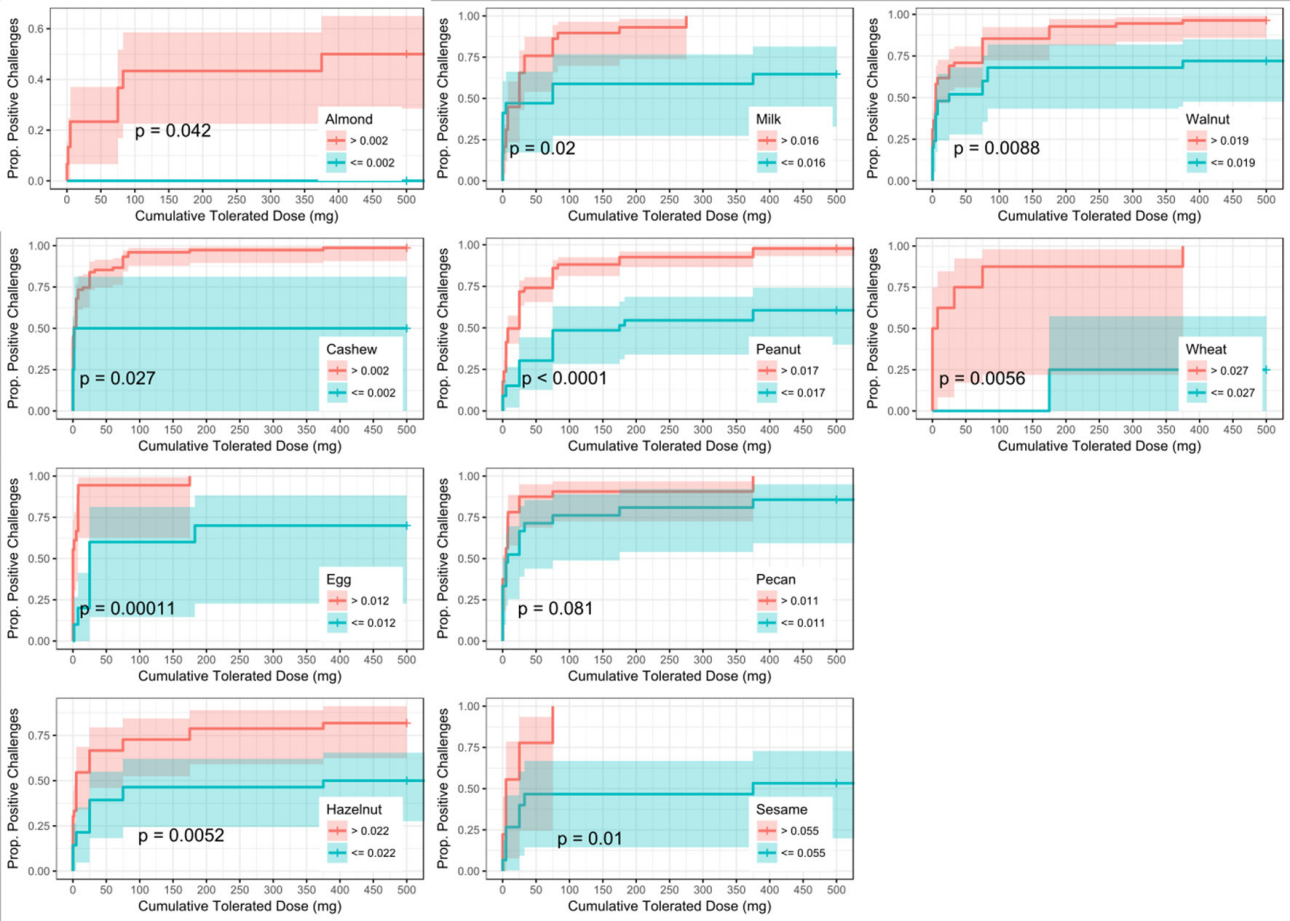
Dose to positive challenge by allergen-specific IgE to Total IgE ratio (sIgEr) threshold and food. Kaplan-Meier curves of dose to positive challenge stratified by the CTD-dependent ROC thresholds for sIgEr by food. Red lines indicate risk of a positive challenge if sIgEr is above the threshold, while blue lines indicate risk for participants with sIgEr at or below the threshold.

Six of the ten derived sIgEr thresholds had AUCs above 0.80, with a lowest AUC of 0.76. At defined values SPT had the best predictive value compared to sIgE and sIgEr. The PPV for all tested foods was 1 except for pecan, which was 0.95. Within sIgE values, sesame was the lowest at 0.64. The sIgEr had a PPV range of 0.68 to 1 with almond having the lowest PPV (Table 6). As with SPT and sIgE, participants with sIgEr values below the threshold were less likely to have a positive challenge at the same CTD as someone with a value above the threshold (Figure 6). Significant risk stratification of food-specific challenge outcome by biomarker threshold was found in the majority of foods (Figures 4–6).

**Figure 7:**
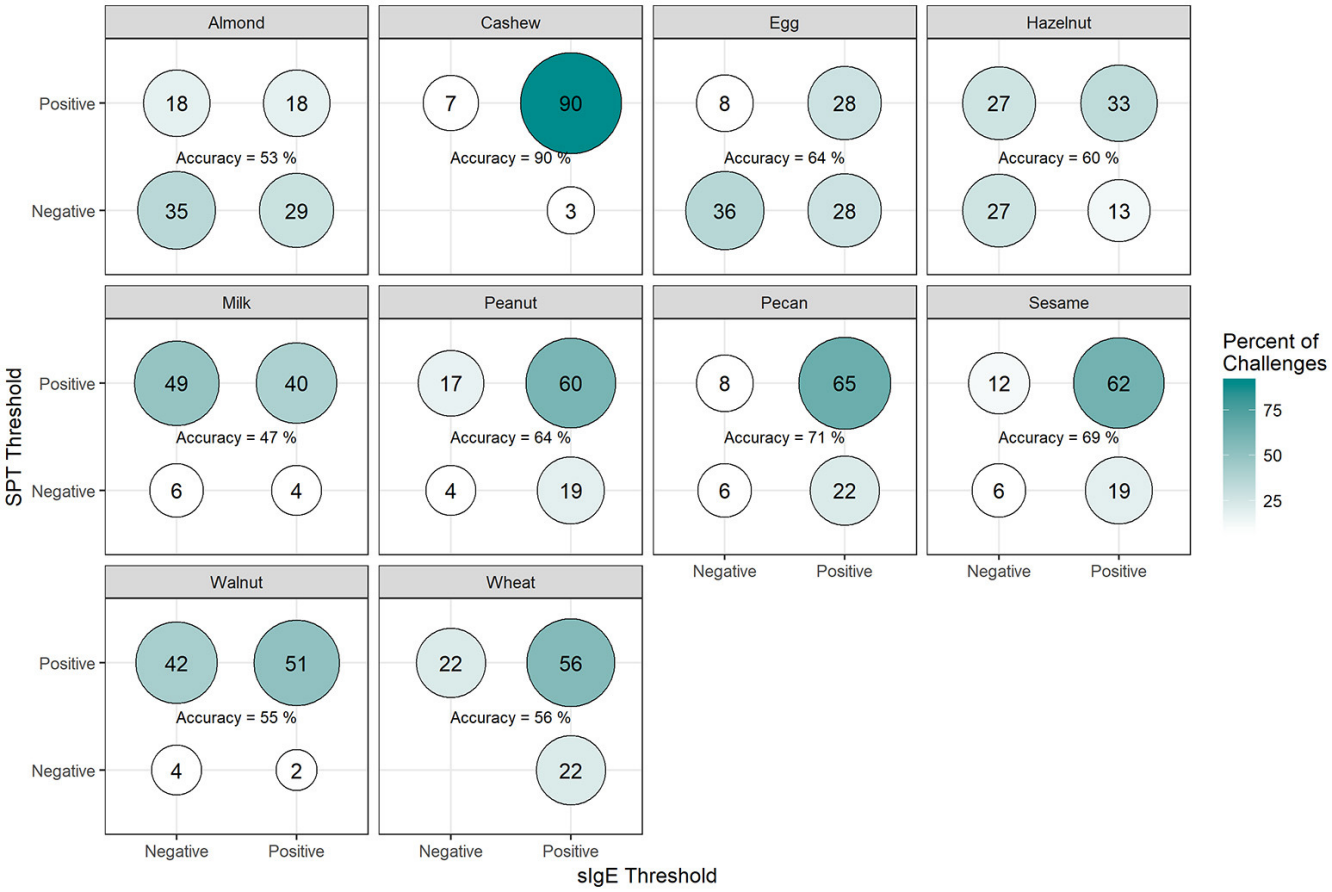
Concordance of skin prick test (SPT) and allergen-specific IgE (sIgE) CTD-dependent thresholds in positive challenges. Among participants with positive challenges, the percentage of participants with each combination of SPT and sIgE values above or below the CTD-dependent ROC thresholds by food. Percentages in each food add up to 100%. Accuracy is the percentage of SPT positive and sIgE positive plus the percentage of SPT negative and sIgE negative.

**Figure 8:**
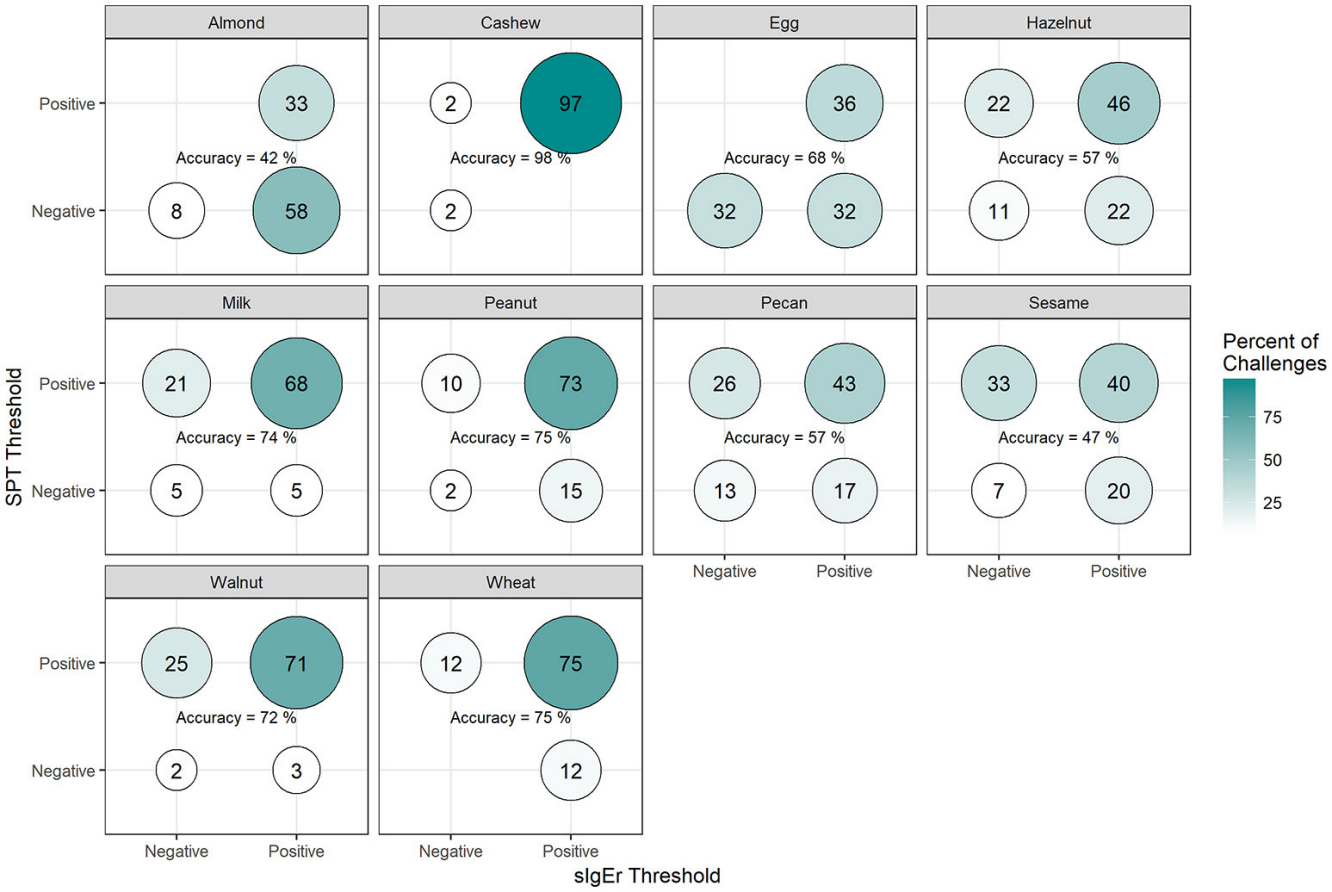
Concordance of skin prick test (SPT) and allergen-specific IgE to Total IgE ratio (sIgEr) CTD-dependent thresholds in positive challenges. Among participants with positive challenges, the percentage of participants with each combination of SPT and sIgEr values above or below the CTD-dependent ROC thresholds by food. Percentages in each food add up to 100%. Accuracy is the percentage of SPT positive and sIgEr positive plus the percentage of SPT negative and sIgEr negative.

Among positive challenges, at least 60% of participants had SPT and sIgE values above the reported CTD-dependent thresholds for four of the ten allergens (cashew, peanut, pecan, and sesame), of which cashew displayed the highest level of SPT and sIgE threshold concordance at 90% (Figure 7). Among almond, egg, and wheat where accuracy was low, the SPT threshold was more likely to be negative when the sIgE threshold was positive. However, among milk and walnut, the SPT threshold was more likely to be positive when the sIgE threshold was negative. The overall agreement of SPT and sIgE thresholds was 65%. Half of the concordance rates for SPT and sIgEr were higher than those calculated for SPT and sIgE (Figure 8). The overall agreement of SPT and sIgEr thresholds was higher than that of SPT and sIgE with 72%.

## Discussion

Presently, the gold standard for confirming food allergy remains the DBPCFC, especially in the research setting; however, the procedure can be time consuming, resource intensive, and carries the risk of life-threatening anaphylaxis (9, 10, 26, 27, 37). Recent studies have shown 40–70% of food allergic patients are allergic to more than one food (2), resulting in the need for multiple food challenges to prove or disprove each allergy. Additionally, positive reactions to placebo are not uncommon and can have a varied clinical presentation. In our experience, 12.7% of participants had positive placebo challenges, which is consistent with the published literature (38–44). In light of these significant burdens, there is a great need for a reliable method of diagnosing food allergies without food challenges, in addition to the ability to stratify participants according to potential risk in scenarios where a food challenge cannot be avoided.

Our large dataset of 1247 baseline OFCs allowed us to evaluate CTDs across several allergens and examine the utility of SPT, sIgE, sIgEr, and a combination of these markers in the prediction of food challenge outcome. SPTs and sIgE remain among the most widely used diagnostic markers for the evaluation of a suspected food allergy due to their simplicity and safety, with SPT providing nearly immediate results. Previous literature reports threshold values for each of these markers with high PPVs in the prediction of food challenge outcome and true food allergy (11, 45–50). We implemented similar methods to those described in the literature to derive optimal thresholds of SPT and sIgE for each individual allergen in our dataset. We further derived thresholds for the ratio of sIgE to tIgE to account for relative proportions of each allergen-specific IgE, which has yet to be evaluated in multi-food allergic patients. While a number of our calculated thresholds for SPT and sIgE values appeared to vary in relation to the thresholds at 95% PPV reported in the literature, differences in our cohort may be due to the fact that our participants are multi-food allergic (11, 45, 46, 49).

In addition to their use as individual predictors of food challenge outcome, prior studies have also assessed the utility of a combination of biomarkers (15, 51); however, to our knowledge, this is the first study to evaluate the utility of combining optimized threshold values for SPT with sIgE or sIgEr. While SPT had the highest PPV values compared to sIgE and sIgEr, the combination of SPT cut-off values with those for sIgE or sIgEr resulted in greater AUCs than SPT, sIgE, or sIgEr alone in the prediction of food challenge outcome. While previous data, mostly in the setting of allergen immunotherapy for allergic rhinitis, have demonstrated sIgEr to be promising as a predictive marker for clinical outcome (52–56), the ratio may have underperformed in our population due to limitations in the number of participants with both sIgE and tIgE values.

The methodology described above was also used in evaluating the association between specific allergens, baseline participant characteristics, and food challenge outcome. Our findings indicate that CTDs vary by allergen, suggesting that the use of identical dosing strategies for food challenges across all may not be the optimal, safest approach. Within our dataset, 50% of our participants had reactions before reaching the 10 mg dose for all foods, exluding almonds. When designing clinical trials that include food challenges, smaller incremental dose steps below 10 mg may aid in reducing the severity of reactions.

Additional findings from our dataset suggest that participants with a history of AD have an increased risk of a positive challenge outcome compared to those without a history of AD. While the presence of AD is often associated with a high rate of false-positives during food allergy testing, especially in children (57–59), our data suggests that among participants who are sensitized to one or more foods, those with a history of AD actually have a higher risk of a positive food challenge than those without a history. This is consistent with the current theory that the impaired skin barrier observed in those with AD may facilitate sensitization through environmental exposure to food allergens, and combined with avoidance of regular oral exposure, lead to true food allergy (60, 61). Although, previous literature has found asthma to be a significant predictor of severe reactions (51, 62), our data did not find asthma to be a significant factor associated with positive challenge outcome. Some studies have shown that age can affect IgE and SPT cutoff levels (22, 63, 64), with lower cutoffs typically used in children < 2 years of age (65, 66), however our analysis did not reveal strong associations with age, SPT/sIgE/sIgEr cutoff levels and challenge outcomes. This is likely due to the limited number of participants aged < 2 years who were challenged in our cohort.

Other studies have similarly explored factors in optimizing predictive outcome. In a retrospective study, DunnGavin et al. used a prognostic model that incorporated gender, age, and prior history of reaction in addition to sIgE, tIgE minus sIgE, and SPT. Their model accurately predicted OFC results 92% percent of the time (67). Cianferoni et al. conducted a retrospective chart review and used a multilogistic regression and discovered that age and history of prior non-cutaneous reactions, when combined with patient's SPT wheal size were predictive of multisystem reactions during food challenges. Simberloff et al. designed and implemented a Standardized Clinical Assessment and Management Plan (SCAMP) to improve sIgE and SPT thresholds to determine which patients would benefit from an OFC. While most studies for food allergy are focused on predictive models to distinguish between a positive or negative food challenge (10, 39, 68), our model also attempts to predict the dose at which a reaction may occur based on biomarkers. We utilized a novel approach to integrate the CTD with food challenge outcome when deriving optimally predictive SPT and sIgE threshold values. Our group has previously found this approach of adjusting for dose to be important in predicting OFC outcomes (62). The primary focus of our analysis was to determine whether the addition of CTD data with food challenge outcome would improve the diagnostic accuracy, as measured by AUC, of derived threshold values for available biomarkers when compared to a logistic ROC approach utilizing food challenge outcome alone. Our analysis did not reveal a statistical difference between these two approaches; however, incorporating CTD into the challenge outcome did allow for risk stratification and the generation of separate Kaplan–Meier curves for individuals with biomarker values above and below the generated thresholds, thus enabling a prediction of the cumulative protein dose that the individual will react to based on biomarker values (Figures 4–6). These findings are clinically useful, especially in the research setting, in that biomarker values above the threshold were associated with a positive outcome at a lower dose compared to those with biomarker values at or below the threshold. For instance, 50% of participants with an almond sIgE > 12.2 kU/L had a positive challenge by 5 mg CTD, compared to only about 16% of participants with almond sIgE < 12.2 kU/L. Therefore, during an oral challenge, clinicians may incorporate smaller dose increments during the early phase of a challenge for a participant with an sIgE above 12.2 kU/L compared to those below.

The results of our study are strengthened by the large sample size of included food challenges and our novel approach in calculating biomarker thresholds using dose-dependent ROC methodology. To qualify for certain trials the level of SPT and/or sIgE had to meet a certain threshold. Our cohort represents a highly allergic subset with high sIgE and SPT measurements, with values higher than what is typically encountered in the average clinical setting (15) but consistent with the baseline characteristics of patients in the research setting (69–71). sIgE values were capped at 101 kU/L, thus adding additional risk of skewing the sIgE and sIgEr to be falsely low. The thresholds reported in our analysis, though generally consistent with the previously reported thresholds in the literature, are relatively high for SPT, sIgE, and their combination (51); however, given the relatively high AUC levels for the majority of the reported individual and combined threshold values, the thresholds may be a reliable marker to use in clinical trials. In such a setting, the promising AUC levels may provide enough confidence to forego the need for food challenges in confirming allergy and determining study eligibility for a subset of participants. Some limitations of the study include the small sample size for several of the allergens (almond, sesame, and wheat). The results reported here should only be considered as hypothesis-driving and need to be validated in future studies involving larger trials.Our novel approach of utilizing CTD-dependent ROC to develop clinical thresholds was not statistically different than the more commonly used approach of thresholds calculated from logistic ROC; however, CTD-dependent approach allows for risk stratification and for predicting the challenge outcome based on biomarker values. Additionally, having multiple food allergies as well as a history of AD appears to increase the risk of a positive outcome during food challenges. The proposed thresholds may not be applicable for participants with biomarker values falling below the cut-off, and, thus, food challenges may still be unavoidable for such patients. There continues to be a need for newer biomarkers, such as BATs, component result diagnostics, and epigenetic markers, or combinations of these, which may be predictive tools across all allergens and should be considered in future studies.

## Conclusion

For the diagnosis of true food allergy, an exact algorithm for determining when an OFC should be performed has yet to be found. Despite remaining the gold standard, food challenges demand significant time and resource requirements and place patients at risk for severe adverse events. As such, dedicated efforts have been made to identify alternative methods of diagnosis. Through our analyses of a large population of standardized food challenges across 11 different foods, we present SPT and sIgE values that are highly predictive of a positive challenge, suggesting food challenges may be unnecessary in the subset of patients with values falling above our reported cut-offs. Additionally our method allows for risk stratification to better predict the dose at which there may be a positive outcome based on biomarker values. While continued efforts will be needed to further refine and identify markers and diagnostic methods outside SPT and sIgE values that are able to fully replace the challenges used today, the ability to potentially forego challenges in the described subset of patients using readily obtainable biomarkers may be an improvement over the current standard of challenges for all patients participating in research.

## Data availability statement

Data are available on request.

## Ethics statement

This study was carried out in accordance with the recommendations of ICH/GCP/CFR guidelines by the Stanford IRB with written informed consent from all subjects. All subjects gave written informed consent in accordance with the Declaration of Helsinki. The protocol was approved by the Stanford IRB.

## Author contributions

Study was designed by SSi, AJL, NP, KN and RSC; study was conducted by SSi, MW, AJL, DK, KN KO, RSC, AA, JP, JS, JT, ST, and JW; data collection and critical review of manuscript was conducted by AA, JP, JS, JT, ST, JW; data collection and analysis was conducted by MC, SS, SSi, NP, SA, DT, KN, and RSC; manuscript was written by SSi, AJL, NP, SJG, KN, and RSC.

### Conflict of interest statement

The authors declare that the research was conducted in the absence of any commercial or financial relationships that could be construed as a potential conflict of interest.
