# Corrigendum: Analysis of a Large Standardized Food Challenge Data Set to Determine Predictors of Positive Outcome Across Multiple Allergens

**DOI:** 10.3389/fimmu.2020.625796

**Published:** 2020-11-30

**Authors:** Sayantani Sindher, Andrew J. Long, Natasha Purington, Madeleine Chollet, Sara Slatkin, Sandra Andorf, Dana Tupa, Divya Kumar, Margaret A. Woch, Katherine L. O’Laughlin, Amal Assaad, Jacqueline Pongracic, Jonathan M. Spergel, Jonathan Tam, Stephen Tilles, Julie Wang, Stephen J. Galli, Kari C. Nadeau, R. Sharon Chinthrajah

**Affiliations:** ^1^ Sean N. Parker Center for Allergy and Asthma Research, Stanford University School of Medicine, Stanford, CA, United States; ^2^ Department of Pharmacy, Lucile Packard Children’s Hospital Stanford, Stanford, CA, United States; ^3^ Department of Medicine, School of Medicine, Stanford, CA, United States; ^4^ Division of Allergy and Immunology, Cincinnati Children’s Medical Center, Cincinnati, OH, United States; ^5^ Division of Allergy and Immunology, The Ann and Robert H. Lurie Children’s Hospital of Chicago, Chicago, IL, United States; ^6^ Division of Allergy and Immunology, The Children’s Hospital of Philadelphia Department of Pediatrics, Perelman School of Medicine at University of Pennsylvania, Philadelphia, PA, United States; ^7^ Division of Clinical Immunology and Allergy, Children’s Hospital Los Angeles, Los Angeles, CA, United States; ^8^ ASTHMA Inc. Clinical Research Center, Northwest Asthma and Allergy Center, University of Washington, Seattle, WA, United States; ^9^ Division of Allergy and Immunology, Department of Pediatrics, Icahn School of Medicine at Mount Sinai, New York, NY, United States; ^10^ Department of Pathology, Stanford University School of Medicine, Stanford, CA, United States; ^11^ Department of Microbiology and Immunology, Stanford University School of Medicine, Stanford, CA, United States

**Keywords:** food challenge, cumulative tolerated dose, AUC, biomarker evaluation, time-dependent ROC

In the original article, there was an error in the section “CTD-Dependent ROC for Clinical Thresholds,” Paragraph 3. The word “sesame” in this sentence is incorrect: “At defined values SPT had the best predictive value compared to sIgE and sIgEr. The PPV for all tested foods was 1 except for sesame, which was 0.95. Within sIgE values, sesame was the lowest at 0.64.”

A correction has been made to the above sentence, as follows: “At defined values SPT had the best predictive value compared to sIgE and sIgEr. The PPV for all tested foods was 1 except for pecan, which was 0.95. Within sIgE values, sesame was the lowest at 0.64.”

The authors apologize for this error and state that this does not change the scientific conclusions of the article in any way.

